# Development and randomized controlled trial of an animated film aimed at reducing behaviours for acquiring antibiotics

**DOI:** 10.1093/jacamr/dlab083

**Published:** 2021-06-17

**Authors:** Sarah Wilding, Virpi Kettu, Wendy Thompson, Philip Howard, Lars J C Jeuken, Madeleine Pownall, Mark Conner, Jonathan A T Sandoe

**Affiliations:** 1 School of Psychology, University of Leeds, Leeds, UK; 2 Kettu Studios, Skipton, UK; 3 Division of Dentistry, University of Manchester, Manchester, UK; 4 School of Healthcare, University of Leeds, Leeds, UK; 5 Leeds Teaching Hospitals NHS Trust, Leeds, UK; 6 School of Biomedical Sciences, University of Leeds, Leeds, UK; 7 Leeds Institute of Medical Research, School of Medicine, University of Leeds, Leeds, UK

## Abstract

**Background:**

Antimicrobial resistance (AMR) is a global health crisis but reducing antibiotic use can help. Some antibiotic use is driven by patient demand.

**Objectives:**

To develop an intervention to discourage antibiotic-seeking behaviour in adults.

**Methods:**

Literature reviewed to identify behaviours for acquiring antibiotics among adults in the community. Behaviour change wheel approach was used to select the target behaviour and behaviour change techniques. An intervention in the form of a short animated film was developed and its potential impact evaluated in a randomized, controlled, online questionnaire study.

**Results:**

Asking a general medical/dental practitioner for antibiotics was identified as the target behaviour. A short stop-motion animated film was chosen to deliver several behaviour-change techniques. Education and persuasion were delivered around information about the normal microbial flora, its importance for health, the negative effect of antibiotics, and about AMR. 417 UK-based individuals completed the questionnaire; median age 34.5 years, 71% female, 91% white ethnicity. 3.8% of participants viewing the test film intended to ask for antibiotics compared with 7.9% viewing the control film. Test film viewers had significantly higher knowledge scores. At 6 week follow up, knowledge scores remained significantly different, while most attitude and intention scores were not different.

**Conclusions:**

Some patients continue to ask for antibiotics. The film increased knowledge and reduced intentions to ask for antibiotics. At 6 weeks, knowledge gains remained but intentions not to ask for antibiotics had waned. Evaluation in the clinical environment, probably at the point of care, is needed to see if antibiotic prescribing can be impacted.

## Introduction

Antimicrobial resistance (AMR) is a global health crisis that requires urgent action.^1^^,^^2^ Although the relationship between antibiotic use and AMR is not straightforward, it is generally considered that more prescribing leads to more resistance.^3^ Most antibiotic prescribing in humans occurs in primary health care, so it is logical to target this setting for interventions to improve prescribing. Antibiotic prescribing is a highly complex process; a recent umbrella review identified 30 broad categories of factors that influence prescribing behaviour and ‘patient influence’ was among the most frequent,^4^ with some antibiotic use driven by patient demand.^5^ A systematic review of studies of interventions aiming to improve the public’s awareness about AMR and behaviours associated with prudent use of antimicrobials found only 20 studies that fulfilled the inclusion critieria.^6^ The interventions included multimodal mass media interventions (including radio, television, cinema, newspapers, bill boards, bus tails, magazines, websites and printed resources such as posters and leaflets); as well as a variety of school-based interventions (educational and printed materials).^6^ A large proportion of the included studies were aimed at school children or parents.^6^ Only one study was not rated as having a high risk of bias and this study involved a verbal presentation to parents with distribution of written materials, which significantly improved knowledge, but did not target any specific behaviour.^6^ Nine of the reviewed studies targeted, or sought information about, specific behaviours, which included attending a doctor’s surgery for a cold,^7^ taking antibiotics for a cold/flu,^8^^,^^9^ seeing a paediatrician,^10^ seeing another clinician if antibiotics were not prescribed,^11^ using alcohol hand sanitizers,^12^ uptake of influenza vaccine,^12^ and purchasing antibiotics without a prescription.^13^^,^^14^ Among studies that targeted behaviours in the UK, posters presented in newspapers and magazines as part of an antibiotic awareness campaign had little impact on people’s attitudes and intentions.^15^ In general, these interventions have not been considered effective in communicating about AMR.^16^ Although global and local UK AMR strategies include public and professional awareness-raising and educational activities as key areas, the most effective ways of intervening with patients and the public to reduce unnecessary antibiotic use are not known.^17–19^ Interventions are often developed without an underpinning theory of behaviour change and without any evidence that they are likely to have the desired outcome.^20^

Although changing human behaviour around antibiotic use is a complex challenge, the behavioural and social sciences offer a range of theories, frameworks, methods and evidence-based principles that can help inform the design of behaviour-change interventions.^21^ The behaviour change wheel is one example of a framework developed to promote a structured approach to intervention design based on theory and evidence.^22^ Improved involvement of patients in shared decision-making (including through educational interventions to change knowledge, attitudes and intentions) has also been shown to reduce use of antibiotics.^23^^,^^24^ The Theoretical Domains Framework (TDF) is a theoretical lens through which to view the cognitive, affective, social and environmental influences on behaviour.

The aim of this project was to develop an intervention aimed at reducing behaviours for acquiring antibiotics among adults in the community with non-serious infections, including evaluation of its impact on their knowledge, attitudes and intentions relating to antibiotics and based on behaviour-change science.

## Methods

Intervention development was based on the behaviour-change wheel approach.^22^ The intervention was developed in three stages (see Figure S1, available as Supplementary data at *JAC-AMR* Online): (1) understanding the behaviour, identifying intervention options, identifying content, and implementation options; (2) intervention planning and production; and (3) evaluation of the intervention. Reporting of the evaluation was undertaken according to the Template for Intervention Description and Replication (TIDieR) checklist.^25^

### Stage 1: Understanding the behaviour

The target population, context and target behaviour were set following a literature review undertaken to identify patient behaviours for acquiring antibiotics (see Supplementary data section 1 for search strategy). Candidate behaviours were tabulated (Table 1) and characterized by their context and the setting and geographical location in which they had been described. The target behaviour was then selected by assessing: relevance to National Health Service (NHS) primary care in the UK (potential impact), potential for modification in practical terms, positive impact on other related behaviours and it had to be measurable.^22^ The potential system of related behaviours surrounding the target behaviour^22^ was discussed within the research group. The research team then identified what needed to change from the literature and through discussion. Factors that needed to change were categorized using the Capability-Opportunity-Motivation-Behaviour (COM-B) model and TDF and intervention functions were derived using Table 2.2 in the Behaviour Change Wheel (BCW) guide to designing interventions.^22^ Identification of potential behaviour-change techniques for the target behaviour was undertaken using the Theory & Techniques Tool (see Table 2).^26^^,^^27^ The APEASE criteria (Affordability, Practicality, Effectiveness and cost-effectiveness, Acceptability, Side-effects/safety, and Equity) were used to inform which Behaviour Change Techniques (BCTs) were feasible and practical to deliver in a primary care setting.^22^

**Table 1.** Patient behaviours for acquisition of antibiotics identified from literature review

Patient behaviour and context	Country	Reference
*Behaviours undertaken at home or within local community*		
Buying antibiotics without a prescription (shop or pharmacy) for self-medication	China, Tanzania, Jordan, Saudi Arabia, Mexico, Bosnia and Herzegovina, Sri Lanka, Jordan	^43–52^
Buying antibiotics without a prescription (shop or pharmacy) in another country and importing for self-medication	UK, USA, Jordan	^45^ ^,^ ^48^ ^,^ ^53^
Keeping leftover antibiotics from a previous personal prescription	Qatar, United Kingdom, France, Belgium, Italy, Spain, Turkey, Thailand, Morocco, and Colombia	^51^ ^,^ ^54–56^
Re-using a previous prescription to obtain antibiotics from a pharmacy	China	
Self-medicating with leftover antibiotics from a previous personal prescription	Jordan, USA, Singapore, Jordan	^45^ ^,^ ^56–59^
Sharing antibiotics with family/friends/social network	Jordan, Saudi Arabia, Qatar, Singapore, Jordan	^45^ ^,^ ^46^ ^,^ ^54^ ^,^ ^56^ ^,^ ^59^
Obtaining antibiotics from ‘black market’ for self-medication	USA	^56^
Obtaining antibiotics from family/friends/social network for self-medication	USA	^56^ ^,^ ^57^
*Behaviours undertaken during consultation with medical professional*		
Requesting an antibiotic from a prescribing healthcare provider	China, USA, UK	^29^ ^,^ ^51^ ^,^ ^57^ ^,^ ^60–62^
Suggesting a diagnosis to a doctor [that implies a need for antibiotics] ‘candidate diagnosis’	USA	^61^
Describing a set of symptoms specifically indexing a particular diagnosis ‘implied candidate diagnosis’	USA	^61^
Exaggerating severity of illness	USA, UK, France, Belgium, Italy, Spain, Turkey, Thailand, Morocco, and Colombia	^55^ ^,^ ^61^
Seeing another doctor if antibiotics not prescribed	Singapore	^59^

**Table 2.** Identifying what needs to change to discourage patients from asking for antibiotics during a general medical/dental consultation, mapping to COM-B component and to theoretical domains framework, intervention ‘function’ and components

What needs to change?	COM-B model component	Theoretical Domains Framework	Intervention function (BCW)	Intervention component/content	BCT (using 93 BCT taxonomy v1)^33^
Lack of awareness of the benefits of avoiding antibiotics	Capability (psychological) Beliefs about what is good—motivation (Reflective)	Knowledge Beliefs about consequences	Education Enablement	Provide information: About the existence of normal the flora (‘helpful bacteria’) and their importance for health. That avoiding antibiotics reduces damage to ‘helpful bacteria’.	5.1 Information about health consequences
Lack of awareness of the normal bacterial flora (‘helpful’ bacteria) living in the body that are important for health	Capability (Psychological)	Knowledge	Enablement	Provide information: On ‘helpful bacteria’. That antibiotics increase the risk of AMR infections.	5.1 Information about health consequences
Lack of awareness about the effects of antibiotics on our helpful bacteria	Capability (Psychological)	Knowledge	Enablement	Provide information: Antibiotics damage normal flora (‘helpful bacteria’) as well as treating bacteria causing infection. Antibiotics increase the risk of AMR infections.	5.1 Information about health consequences
Belief that antibiotics do not affect the body’s helpful bacteria and do not lead to AMR	Motivation (Reflective)	Beliefs about consequences	Persuasion (create negative feelings about asking for antibiotics)	Provide information that antibiotics increase the risk of AMR infections through their effect on the body’s normal flora and that avoiding antibiotics reduces this risk	5.1 Information about health consequences
Lack of acceptance that AMR is relevant/bad	Motivation (Reflective)	Beliefs about consequences	Education Persuasion (create negative feelings about asking for antibiotics)	Create a sense of personal jeopardy by using language in the second person to explain how AMR occurs, is of personal relevance and is a bad thing. Use visual imagery to evoke fear of AMR (skull image Figure S2 and colour red).	5.2 Salience of consequences
Lack of concern about antibiotic resistance	Motivation (Reflective)	Beliefs about consequences	Education	Provide information about how the risk of AMR can be avoided	5.2 Salience of consequences
Incorrectly believing that antibiotics are effective/ necessary for non-severe infections	Motivation (Reflective)	Beliefs about consequences	Education Persuasion (create negative feelings about asking for antibiotics)	Provide reassurance that not all infections require antibiotic treatment and you may feel better without antibiotics	5.6 Information about emotional consequences
Lacking trust in healthcare professional’s judgement	Motivation (reflective)	social/professional role and identity	Persuasion	Provide reassurance that clinicians know when to use antibiotics	5.6 Information about emotional consequences
Lack of concern about having an adverse antibiotic reaction during treatment	Motivation (Reflective)	Beliefs about consequences	Persuasion (create negative feelings about asking for antibiotics)	Provide information on antibiotic side effect/harms	5.1 Information about health consequences
Trust in health care providers’ advice if they don’t prescribe antibiotic	Motivation (Automatic)	Reinforcement	Persuasion (create positive feelings about advice not to take antibiotics)	Provide reassurance that GP/GDP will prescribe antibiotics if they are clinically indicated	5.1 Information about health consequences
Intention to avoid antibiotics if possible	Motivation (Reflective)	Intention	Persuasion	Provide encouragement that it is OK to avoid antibiotics if a GP/GDP advises this	5.6 Information about emotional consequences
Sufficient time to discuss antibiotic harms versus benefits with a doctor/dentist	Opportunity (physical)	Environmental context and resources	Enablement (allow/encourage discussion about avoidance of antibiotics)	Time in consultation. (Design intervention to minimally impact on consultation time but promote discussion.)	12.2 Restructuring of social environment (this was not considered to be feasible)

AMR, antimicrobial resistance; AMR, antimicrobial resistance; BCW, behaviour change wheel; GP, general practitioner; GDP, general dental practitioner; BCT, behaviour change techniques.

The research team identified by consensus the target population as adults in the community who were obtaining antibiotics for their own use. We considered the situation where an individual was seeking antibiotics for another person (e.g. a child) and concluded this to be a more complex situation, likely to need a different intervention, and excluded this behaviour. For the literature review, we excluded studies including patients with sexually transmitted infections, cystic fibrosis and tuberculosis, as these situations have existing specialist support structures. We excluded studies on people seeking antiviral and antiparasitic medication. Finally, we excluded studies of patients taking ‘rescue’ antibiotics e.g. in the context of chronic obstructive pulmonary disease, where this was part of a plan agreed with a healthcare professional. 823 articles were identified, of which 25 fulfilled the inclusion criteria. The candidate behaviours to acquire antibiotics identified are shown in Table 1. The target behaviour (Figure S2) was adult patients asking for antibiotic treatment for themselves during a consultation with a general medical (GP) or dental practitioner (GDP). In terms of what needed to be done differently, we wanted to discourage people from pressurizing their practitioner by asking for antibiotics. We concluded that the system of behaviours within which the target behaviour occurred included: patients seeking and attending a medical/dental consultation and, choosing not to self-medicate at home. Although studies have investigated patients’ expectations or desire for antibiotics,^6^^,^^28–30^ the actual behaviour of asking for antibiotics was much less frequently investigated.^5^ Relatively high rates of patients asking for antibiotics of 20%–26% have been described.^31^^,^^32^ We found no information in the literature about motivating change in the target behaviour through explanation of the potential health benefits of avoiding antibiotics. We hypothesized that this behaviour might be a novel means of bringing about a change in antibiotic use. The appropriate behaviour-change techniques were identified as: providing information about health consequences, salience of consequences, and emotional consequences^33^ with anticipated impact on beliefs about consequences, by a mechanism of reflective motivation.^22^ Mapping of the barriers to change to the TDF and associated BCTs is shown in Table 2.

### Stage 2 intervention (animation) planning and production

Principles were developed for the animation and intervention components identified in stage 1. We also consulted experts outside our research team including those in infection control and public engagement. The animation was then designed to incorporate these intervention components. Initially, a storyboard was drafted and modified on paper. A transcript was written and modified iteratively with feedback from the research team. An ‘animatic’ (a digital merging of storyboard drawings and voiceover to give a real-time digital overview of the film) was created. This was shared with the research team by e-mail and face-to-face meetings and modified iteratively. Design took into consideration the characteristics of an animation that can optimize learner understanding and cognitive theory of multimedia learning.^5^ Colour was used carefully, but with a ‘Western’ perspective e.g. red to evoke fear and blue to indicate trustworthiness. Once the animatic had been agreed, the animation was built using a mixture of plasticine/silicone models, stop frame animation filming and digital animation.

A stop frame animation was chosen as the initial intervention to develop and assess, because it could deliver many of the BCTs identified as influencing the target behaviour (Table S1), could be used to engage emotions, and was considered by the research team to be acceptable, practicable, potentially effective/cost-effective, affordable and safe, and could deliver the intervention in an equitable manner. Animated films are a good potential means to communicate difficult subjects in an easily understandable form.^34^ The characteristics of an animation that can optimize learner understanding take into account cognitive theory of multimedia learning.^34^ Animations have a large measurable impact on remembering information, particularly if they are presented in a fun, non-threatening and interesting format. Principles for the animation and key messages, summarizing the intervention components are shown in Table S2. Although imagery intended to evoke fear of AMR was incorporated (Figure S3), positive messages about the benefits of avoiding antibiotics were also included. The final transcript is in Box S2. Important changes to the transcript during the iterative refinement included: to use the term ‘helpful’ rather than ‘healthy’ bacteria; to use the term ‘fight infection’; and to emphasize the role of the doctor or dentist. In addition, the emphasis was changed to ‘you’ rather than a collective ‘us’ throughout the animation, to highlight the personal jeopardy of AMR.^16^ The final message ended up very similar to a previously used slogan ‘Use antibiotics only if a doctor prescribes them’, developed for Spanish speakers in Colorado.^10^ The final intervention comprised, a short (51 second) stop-motion plasticine-based animated film available at https://www.youtube.com/watch?v=r_50QNX0-t0. This was produced in the style of well-known animated characters such as Wallace and Gromit, in an effort to make it entertaining and engaging.

### Stage 3. Evaluation of the impact of the animation on knowledge, beliefs/attitudes and intentions

Stage 3 incorporated a randomized controlled trial design to assess the impact of the animation on beliefs compared with control. Ethics approval was granted by the University of Leeds, School of Psychology Research Ethics Committee (ref: PSC-685; Date: 30.04.19). A sample of UK adults was recruited via Prolific (https://prolific.ac/), an online study recruitment website where participants are paid for taking part in research. Eligible participants were those over 18 currently residing in the UK. Participants were recruited on 31 May 2019 for the Time 1 survey and 12 July 2019 for the Time 2 survey.

On following the link, participants were asked to read information about the study and indicate their consent to take part. They were then individually randomized via Qualtrics (1 : 1) to one of two conditions: shown one of two short animated films (see below) whose content related to AMR (experimental) or the use of proportional representation in Canada (control). The control video was selected as it had been created by the same animator, it was also of a similar duration, and communicated information on a topic of similar complexity. Participants were asked to complete the same questionnaire immediately after watching the film (Time 1) and 6 weeks later (Time 2).

Questions were developed by the research team and were directed to the target behaviour, as well as related behaviours. In addition to these questions, items were presented to assess the knowledge, attitudes, and beliefs about antibiotics. General reactions to the film were assessed using three items (‘I found the video interesting’; ‘I found the video informative’; ‘I found the video entertaining’). Knowledge of antibiotics was assessed using 10 items, separated into three key messages. Message 1 focused on the idea that we have ‘helpful bacteria’ that are important for health (‘All bacteria are bad for us’; ‘There are some good bacteria in our bodies that are important for our health’; ‘Some bacteria can be good for our health’). Message 2 focused on the idea that antibiotics kill our ‘helpful bacteria’ and that this allows resistant bacteria to multiply (‘Antibiotics kill only the bad bacteria that cause illnesses’; ‘Antibiotics kill both good and bad bacteria in our bodies’; ‘Although antibiotics kill the bacteria that make us unwell, they also kill the good bacteria that are important for health’). Message 3 focused on the idea that taking antibiotics when you don't need them can harm your health (‘Antibiotics are always needed to get well’; ‘We should only take antibiotics when recommended by our doctor or dentist’; ‘Antibiotics are not always the best treatment’; ‘Taking antibiotics when not needed might be bad for my long-term health’). Attitudes towards aspects of antibiotic use were tested using eight items, and intentions were assessed using seven items, the wording of the individual items is provided in Table 3. Participants were e-mailed after 6 weeks with a link to the online questionnaire. Participants were paid £3.40 for completing both parts of the survey.

**Table 3.** Results of questionnaire used to evaluate the animation intervention and baseline knowledge, beliefs and intentions toward antibiotic acquisition^a^

	Time 1		6 weeks	
Question	Experiment (*n = *211)	Control (*n = *206)	Experiment (*n = *211)	Control (*n = *206)
Knowledge				
1. Some ‘good’ bacteria are important for health.	4.75 (0.40)^***^	4.48 (0.56)	4.65 (0.49)	4.61 (0.47)
2. Antibiotics kill ‘good’ bacteria.	4.44 (0.79)^***^	3.77 (0.85)	4.31 (0.78)^***^	4.06 (0.76)
3. Taking antibiotics when not needed can harm health.	4.56 (0.50)^**^	4.41 (0.55)	4.56 (0.48)	4.52 (0.48)
Attitudes/beliefs				
4. I am in favour of asking a doctor or dentist for antibiotics if I think I need them.^b^	2.88 (1.25)	2.90 (1.29)	2.78 (1.18)	2.85 (1.23)
5. I expect a doctor or dentist to prescribe antibiotics if I say I need them.	2.25 (1.32)	2.27 (1.25)	2.07 (1.20)	2.16 (1.15)
6. It is best to avoid taking antibiotics unless recommended by my doctor/dentist.	4.77 (0.45)^**^	4.63 (0.59)	4.64 (0.61)	4.60 (0.65)
7. It is important to question your doctor or dentist about whether I really need to take antibiotics.	4.37 (0.62)	4.31 (0.74)	3.85 (0.87)	3.84 (0.85)
8. It is not a good idea to self-medicate on antibiotics (e.g. using up antibiotics left over from a previous course or someone else’s previous treatment).	4.64 (0.76)	4.64 (0.69)	4.64 (0.72)	4.64 (0.75)
9. When prescribed antibiotics by my doctor or dentist, it is always a good idea to ensure you use them all as prescribed, even if you feel better.	4.69 (0.58)	4.63 (0.62)	4.57 (0.74)	4.55 (0.71)
10. Buying antibiotics on the internet in order to treat yourself can be helpful.	1.50 (0.97)	1.67 (1.14)	1.61 (0.97)	1.54 (0.82)
11. I should not expect a doctor or dentist to prescribe antibiotics if they feel I do not need them.	4.55 (0.68)^*^	4.39 (0.85)	4.47 (0.73)	4.45 (0.75)
Intentions				
12. I will not ask my doctor or dentist for antibiotics if I could do without.	4.37 (0.84)^*^	4.15 (1.00)	4.21 (0.89)	4.31 (0.76)
13. I plan to avoid treating myself with antibiotics (e.g., using-up antibiotics left over from a previous course or someone else’s previous treatment).	4.38 (1.09)	4.26 (1.13)	4.43 (0.97)^*^	4.20 (1.23)
14. I intend to buy antibiotics on the internet in order to self-medicate.	1.18 (0.47)	1.27 (0.59)	1.25 (0.56)	1.27 (0.56)
15. I would avoid taking antibiotics unless recommended by my doctor/dentist.	4.62 (0.69)	4.50 (0.78)	4.50 (0.78)	4.51 (0.74)
16. I will question my doctor or dentist about whether I really need to take antibiotics even if they suggest them.	3.38 (1.16)	3.37 (1.11)	3.48 (1.10)	3.41 (1.09)
17. When prescribed antibiotics by my doctor or dentist, I will ensure I take them all as prescribed, even if I feel better.	4.64 (0.64)	4.50 (0.88)	4.54 (0.75)	4.52 (0.76)
18. I will keep any leftover antibiotics I have to use if I need them.	2.80 (1.35)	2.78 (1.39)	1.80 (1.04)	1.65 (0.90)

a Mean (SD) are shown for main measures in the intervention (antibiotic animated film, *N = *211) and control (other animated film, *N = *206) conditions at both timepoints. Note tests of differences between conditions.

* *P < *0.05,

** *P < *0.01,

*** *P < *0.001.

b All questions used the same response item scoring (strongly disagree to strongly agree) and therefore the low scores demonstrate low levels of intention/cognitions relating to antibiotic use behaviour.

Questionnaire items were each rated on a five-point Likert type scale (strongly disagree to strongly agree, scored 1 to 5). For analysis we first tested for any differences between those who only completed the questionnaire at time 1 and those who completed questionnaires at both time 1 and time 2, to test the representativeness of the final sample. We also tested for difference between the two conditions on demographic measures in the final sample to assess the success of the randomization. We then examined reactions to the two videos. Subsequent analyses used ANOVA to assess differences in knowledge, attitude/beliefs and intentions between the intervention and control conditions at Time 1 and at Time 2 (6 week follow up).

## Results

### Stage 3. Animation evaluation

479 participants watched an animated film and completed the questionnaire at Time 1. Of these, 417 participants also completed the questionnaire at Time 2 and their data could be matched across timepoints. For the final sample of 417, the median age was 34.5 years (range 18–74), 71% of the sample were female, 69% were in paid work, and 91% of the sample reported their ethnicity as white.

Participants completing both parts of the survey were significantly older (mean = 36.7 years, SD = 12.9) than those only completing the first part (mean = 32.4 years, SD = 13.6, *P = *0.009). There were no other significant differences between participants completing the Time 1 or both Time 1 and Time 2 follow up parts of the survey (in terms of gender, occupation, socioeconomic status, and ethnicity).

For messages 1, 2 and 3, questionnaire responses were highly intercorrelated (α  = 0.66–0.80) and were averaged. Responses to the attitude, belief and intention questions were not highly intercorrelated and these were analysed individually. The majority of the participants in each group agreed/strongly agreed that the film they watched was informative (experimental condition, *N = *188; 89.1%; control condition, *N = *194; 94.2%) and interesting (experimental condition, *N = *176; 83.4%; control condition, *N = *179; 86.9%). A smaller proportion agreed/strongly agreed that the film was entertaining (experimental condition, *N = *136; 64.4%; control condition, *N = *101; 49.0%).

Results for each questionnaire item are shown in Figure S4. In terms of the intended target behaviour, 87% of participants who had viewed the animated film stated they would not ask for antibiotics (agreed or strongly agreed with the Q12 intention item ‘I will not ask my doctor or dentist for antibiotics if I could do without’), which was higher than the 81% who viewed the control film. 7.9% of control condition individuals indicated that they would ask for antibiotics, versus 3.8% in the experimental condition. Table 3 shows the main analyses testing differences in knowledge, attitudes/beliefs and intentions between the experimental and control conditions at Time 1 and at Time 2 (6 week follow up). At Time 1, participants in the experimental condition reported significantly (*P < *0.01) better knowledge than the control condition about antibiotics in relation to some bacteria being important for health (Q1: message 1), antibiotics killing good bacteria (Q2: message 2), and that taking antibiotics when not needed could harm health (Q3: message 3). By Time 2 only the difference for message 2 remained significant (*P < *0.001), although knowledge scores for all three messages remained higher in the experimental compared with the control condition. Table 3 also shows results of the analyses for the attitude/beliefs and intention (reflective motivation) questions. Here, relatively few significant differences were observed between the two conditions. In relation to attitudes/beliefs, at time 1, only the item (Q6: ‘It is best to avoid taking antibiotics unless recommended by my doctor/dentist’) was significantly more likely to be agreed with in the experimental versus the control conditions. At 6 week follow up, there were no significant differences in the attitude questions. At Time 1, one item (Q11: ‘I should not expect a doctor or dentist to prescribe antibiotics if they feel I do not need them’) was significantly more likely to be agreed with in the experimental versus the control conditions. At Time 2, only the item [Q13: ‘I plan to avoid treating myself with antibiotics (e.g. using up antibiotics left over from a previous course or someone else’s previous treatment)’] was significantly more likely to be agreed with in the experimental versus the control conditions. Both experimental and control conditions agreed it is not a good idea to self-medicate with antibiotics by using up antibiotics left over from a previous course or someone else’s previous treatment or purchased from the internet and did not have plans to acquire antibiotics by these means, and these did not differ on statistical analysis. The CONSORT checklist for the study is available in Figure S5.

## Discussion

Viewing the intervention animation film produced an ∼4% decrease in participants intentions to ask for antibiotics compared with controls. Participants had a lower rate of intention to request antibiotics compared with the 20%–26% reported in previous UK studies of individuals with respiratory tract infections.^31^^,^^32^ Nevertheless, if a baseline of 8% of patients asked a GP for antibiotics and the majority were prescribed, there is potential to reduce a substantial number of prescriptions. Control condition participants demonstrated a high level of knowledge about antibiotics, but under the experimental conditions, the animation film also had a statistically significant effect on: knowledge that there are helpful bacteria; that antibiotics kill helpful bacteria, and taking antibiotics when you don't need them can harm your health. The importance of the normal microbial flora (microbiome) to general health and wellbeing is becoming increasingly apparent, as is the damaging impact of antibiotics on the microbiome,^35^ and this concept was used as part of a persuasive and incentivizing approach to discourage people from asking for antibiotics. The animation was designed to engage at an emotional level and positive messages were combined with imagery intended to evoke fear of AMR.

We checked our choice of target behaviour by asking questions about other behaviours for obtaining antibiotics; most participants agreed that self-medicating with antibiotics was not a good idea, a finding that is consistent with very low (<1%) rates of self-medication in a previous UK study.^32^ There appeared to be a delayed effect of the test animation, in that those who had seen the video were significantly more likely to say they would avoid self-medication with antibiotics at the 6 week follow-up questionnaire. Other related behaviours, not directly targeted by the intervention, such as taking medications as advised by the prescriber were not impacted by the experimental film. The effects of viewing the animated film attenuated over 6 weeks, which may be why it has been hard to demonstrate the effectiveness of public awareness campaigns, as any effects are short-lived. We concluded, like others,^15^ that interventions may be most effective if used at the point of care (e.g. waiting rooms and prior to consultations). We aim to evaluate the intervention in various care settings (including before unscheduled and routine GP and GDP appointments) to identify the context in which it would work best.

Levels of awareness of AMR among the general public have been reported to be variable but are often low.^36–38^ A recent Wellcome Trust report has reinforced the importance of using clear and understandable language in communications about AMR.^16^ A number of short films have been used to inform people about AMR, they differ from the current intervention in being longer, containing much more information and being intended for educational use.^39^^,^^40^ Although education and persuasion concerning AMR were elements of the current intervention, we did not specifically set out to assess knowledge of AMR.

### Limitations

In terms of study generalizability, the majority of participants in the evaluation were female; however, more women are treated with antibiotics than men, and women visit their GP more often than men.^41^^,^^42^ Participants undertaking the questionnaire were well informed about the issues, so further testing in less-well-informed individuals is necessary. The indicated rate of the target behaviour (asking for antibiotics) in the control group was 7.9%, which is lower than the 20%–26% reported in patients with respiratory tract infection, one of the most common reasons for antibiotics to be prescribed,^31^^,^^32^ highlighting the need to evaluate the intervention in a clinical context. Approximately 90% of participants were White, so further evaluation in areas with higher ethnic diversity would be required. The video is likely to need to be combined with other interventions in order to effectively change intentions and behaviours toward antibiotic use.

### Future work

Further research should include testing in a clinical setting (e.g. prior to consultations), exploration of the mechanism of action, and the impact of incorporating it as an element of a complex intervention. Further research should also investigate methods of increasing the influence of the film, e.g. exposure more than once. We plan to investigate the effect of encouraging patients to proactively tell their GP/GDP if they would prefer to manage without antibiotics, thereby substituting the behaviour of asking for antibiotics with an alternative behaviour.

### Conclusions

Some patients continue to ask their doctor or dentist for antibiotics. The animated film developed and tested here showed potential as an intervention to discourage patients from asking for antibiotics. It produced a sustained increase in knowledge but impacts on intentions not to ask for antibiotics had waned at 6 weeks. Evaluation in the clinical environment will be needed to see if these intentions translate into behaviour change and a reduction in antibiotic prescribing.

## Supplementary Material

dlab083_Supplementary_Data
