# Supplementary material for: Development and randomized controlled trial of an animated film aimed at reducing behaviours for acquiring antibiotics
Source: JAC Antimicrob Resist. 2021 Jun 17;3(2):dlab083. doi: 10.1093/jacamr/dlab083 (PMC8251327; doi:10.1093/jacamr/dlab083)
Supplement: dlab083_Supplementary_Data [file dlab083_Supplementary_Data.docx]

**Supplementary data**

**Behavioural Analysis for the animation film aimed at reducing patient requests for antibiotics. Supplementary materials**

Literature review for target behaviour

A search of Pubmed was undertaken (last access 4/5/2020) using search terms "anti-bacterial agents"[MeSH Major Topic], "behavior"[MeSH Major Topic, Title/Abstract]), "patient"[Title/Abstract], "seeking"[Title/Abstract].

Inclusion criteria: Qualitative and quantitative research studies concerned with behaviours relating to the acquisition of antibiotics for self-medication, and objective evidence of the behaviour taking place describing

Exclusion criteria: Adults seeking antibiotics for others (e.g. children); specialist situations: mycobacterial treatment, sexual health clinic, cystic fibrosis; hospital setting; behaviours towards antiviral/antiparasitic treatment; self-medication behaviours for pre-prescribed ‘rescue” antibiotics.

### Table S1. Principles for the animation intervention

| **Principle/objective** | **Key features** |
| --- | --- |
| Guiding principles |  |
| To be entertaining or interesting | Stop frame plasticine-based animation |
| To appeal to a wide audience | Stop frame plasticine-based animation |
| To be gender neutral | Avoid gender references |
| To be race neutral | Avoid race references |
| To be easy to understand | Use plain English |
| To be easy to use | Keep the animation short (<1 minute)  Pace steady  Have both audio and text transcript |
| To be factual | n/a |
| To be convincing | Use a narrator to communicate scientific content  Make characters/content believable |
| To be easily accessed/shared | Accessed on a mobile device, computer |
| Key messages summarising animation intervention elements |  |
| 1. Communicate information about the normal microbial flora and its role in maintaining good health | Provide information about ‘helpful’ bacteria that live inside everyone and that these bacteria that are important for health |
| 1. Communicate that antibiotics have a negative effect on the normal microbial flora | Provide information that antibiotics kill the helpful bacteria that live inside everyone, as well as “bad” bacteria that cause infection.  Provide information about the risk of antibiotic resistant bacteria living inside being increased by antibiotics |
| 1. Communicate the idea that you can look after your helpful bacteria by avoiding antibiotics | Use a “self-care” approach to incentivise patients not to expect/request antibiotics |

Table S2. Demographic breakdown of participants by condition

|  |  | Antibiotic video (n = 211) | | Control (n = 207) | |
| --- | --- | --- | --- | --- | --- |
| Age | Mean (SD) | 36.13 (12.76) |  | 37.27 (12.13) | |
|  |  | N | % | N | % |
| Gender | Female | 154 | 73.0 | 143 | 69.1 |
|  | Male | 56 | 26.5 | 63 | 30.4 |
|  | Non-binary/gender fluid | 1 | 0.5 | 1 | 0.5 |
| Occupation | Work full-time/part-time | 139 | 65.9 | 148 | 71.5 |
|  | In education | 24 | 11.4 | 24 | 11.6 |
|  | Not currently employed | 47 | 22.3 | 47 | 22.7 |
|  | Other | 1 | 0.5 | 2 | 1.0 |
| Ethnicity | White | 196 | 92.9 | 184 | 88.9 |
|  | Mixed/multiple ethnic groups | 5 | 2.4 | 9 | 4.3 |
|  | Black/African/Caribbean/Black British | 4 | 1.9 | 4 | 1.9 |
|  | Asian | 6 | 2.8 | 7 | 3.4 |
|  | Other | 0 | 0.0 | 3 | 1.4 |

Box. Final animation transcript

| “Some bacteria cause infections, but not all bacteria are bad. We all have many helpful bacteria living inside us. These helpful bacteria help us fight infections and keep us healthy. When we take antibiotics our helpful bacteria as well as infecting bacteria are killed, allowing harmful, antibiotic-resistant bacteria to grow inside us. Looking after your own helpful bacteria will keep you well – so only take antibiotics if your doctor or dentist really thinks you need them.” |
| --- |

Figure S1. Steps of methodology for intervention development and evaluation

Stage 2

Stage 3

Stage 1

Stage 3:

Evaluation of the impact of the animation on knowledge, beliefs/attitudes and intentions.

Stage 2:

Intervention (animation) planning and production

Stage 1:

Understanding the behaviour

Literature review

Defining behaviour

Intervention target and method of behaviour change defined via COM-B and TDF frameworks

Intervention evaluation via online survey.

Longitudinal randomised controlled trial to assess impact on cognitions and intention over six-week follow up.

Intervention storyboard and transcript development

Figure S2. Target behaviour key components

**Key factors used to assess target behaviour Target behaviour**

Potential impact: Relevance to National Health Service (NHS) primary care in the UK

Adult patients asking for antibiotic treatment for themselves during a consultation with a general medical (GP) or dental practitioner (GDP

Potential for modification in practical terms

Positive impact on other related behaviours

Measurable

Figure S3. Image from animation


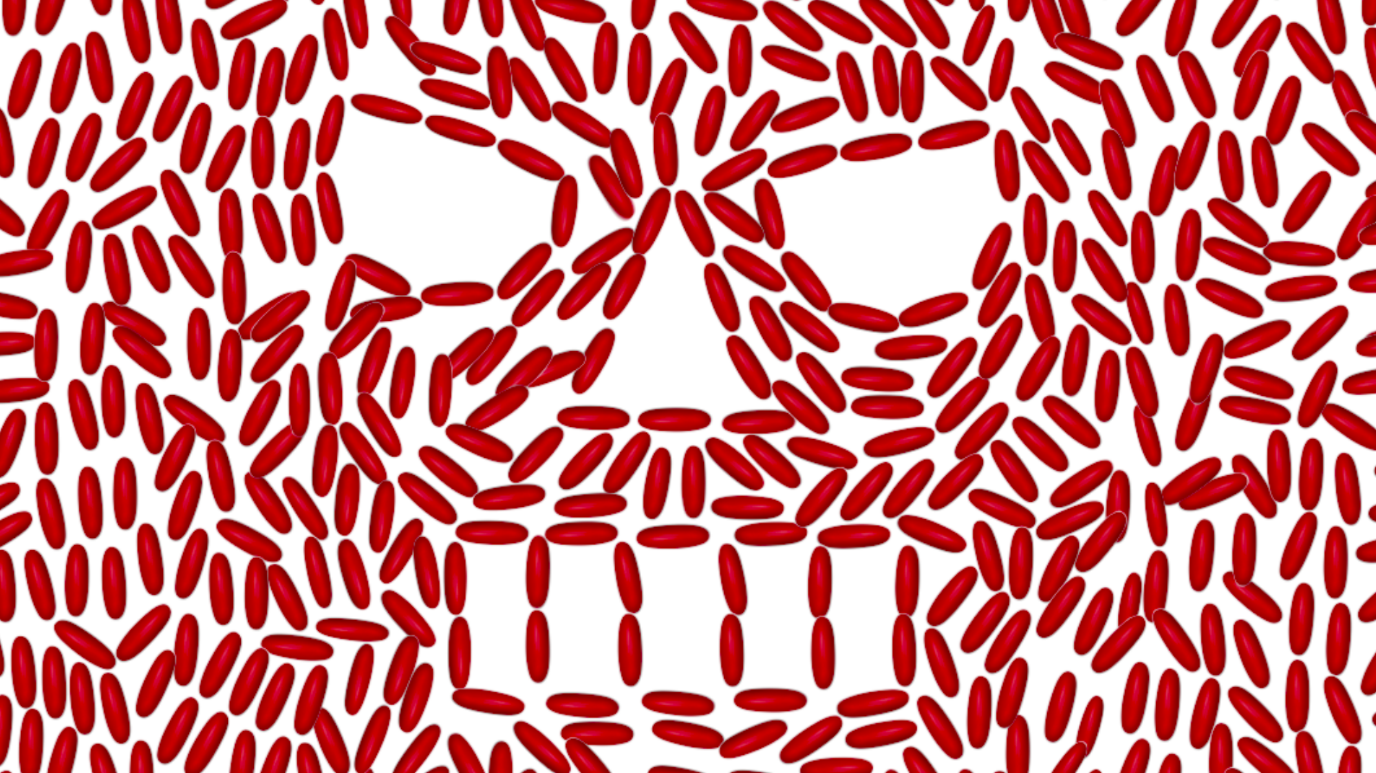


Figure S4 Results of items in the evaluation questionnaire.

Figure S5. CONSORT checklist.

| Section/Topic | Item No | Checklist item | Reported on page No |
| --- | --- | --- | --- |
| Title and abstract | | | |
|  | 1a | Identification as a randomised trial in the title | 1 |
|  | 1b | Structured summary of trial design, methods, results, and conclusions (for specific guidance see CONSORT for abstracts) | 2 |
| Introduction | | | |
| Background and objectives | 2a | Scientific background and explanation of rationale | 3 |
|  | 2b | Specific objectives or hypotheses | 4 |
| Methods | | | |
| Trial design | 3a | Description of trial design (such as parallel, factorial) including allocation ratio | 8 |
|  | 3b | Important changes to methods after trial commencement (such as eligibility criteria), with reasons | NA |
| Participants | 4a | Eligibility criteria for participants | 8 |
|  | 4b | Settings and locations where the data were collected | 8 |
| Interventions | 5 | The interventions for each group with sufficient details to allow replication, including how and when they were actually administered | 6-8 |
| Outcomes | 6a | Completely defined pre-specified primary and secondary outcome measures, including how and when they were assessed | 8 |
|  | 6b | Any changes to trial outcomes after the trial commenced, with reasons | NA |
| Sample size | 7a | How sample size was determined |  |
|  | 7b | When applicable, explanation of any interim analyses and stopping guidelines | NA |
| Randomisation: |  |  |  |
| Sequence generation | 8a | Method used to generate the random allocation sequence | 8 |
|  | 8b | Type of randomisation; details of any restriction (such as blocking and block size) | 8 |
| Allocation concealment mechanism | 9 | Mechanism used to implement the random allocation sequence (such as sequentially numbered containers), describing any steps taken to conceal the sequence until interventions were assigned | 8 |
| Implementation | 10 | Who generated the random allocation sequence, who enrolled participants, and who assigned participants to interventions | 8 |
| Blinding | 11a | If done, who was blinded after assignment to interventions (for example, participants, care providers, those assessing outcomes) and how | 8 |
|  | 11b | If relevant, description of the similarity of interventions | NA |
| Statistical methods | 12a | Statistical methods used to compare groups for primary and secondary outcomes | 10 |
|  | 12b | Methods for additional analyses, such as subgroup analyses and adjusted analyses | 10 |
| Results | | | |
| Participant flow (a diagram is strongly recommended) | 13a | For each group, the numbers of participants who were randomly assigned, received intended treatment, and were analysed for the primary outcome | 10 |
|  | 13b | For each group, losses and exclusions after randomisation, together with reasons | 10 |
| Recruitment | 14a | Dates defining the periods of recruitment and follow-up | 8 |
|  | 14b | Why the trial ended or was stopped | NA |
| Baseline data | 15 | A table showing baseline demographic and clinical characteristics for each group | Table S2 |
| Numbers analysed | 16 | For each group, number of participants (denominator) included in each analysis and whether the analysis was by original assigned groups | Table 3 |
| Outcomes and estimation | 17a | For each primary and secondary outcome, results for each group, and the estimated effect size and its precision (such as 95% confidence interval) | Table 3 |
|  | 17b | For binary outcomes, presentation of both absolute and relative effect sizes is recommended | NA |
| Ancillary analyses | 18 | Results of any other analyses performed, including subgroup analyses and adjusted analyses, distinguishing pre-specified from exploratory | NA |
| Harms | 19 | All important harms or unintended effects in each group (for specific guidance see CONSORT for harms) | NA |
| Discussion | | | |
| Limitations | 20 | Trial limitations, addressing sources of potential bias, imprecision, and, if relevant, multiplicity of analyses | 13 |
| Generalisability | 21 | Generalisability (external validity, applicability) of the trial findings | 13 |
| Interpretation | 22 | Interpretation consistent with results, balancing benefits and harms, and considering other relevant evidence | 13 |
| Other information | | |  |
| Registration | 23 | Registration number and name of trial registry | NA |
| Protocol | 24 | Where the full trial protocol can be accessed, if available | NA |
| Funding | 25 | Sources of funding and other support (such as supply of drugs), role of funders | 15 |
